# Metagenomic analysis of viruses in toilet waste from long distance flights—A new procedure for global infectious disease surveillance

**DOI:** 10.1371/journal.pone.0210368

**Published:** 2019-01-14

**Authors:** Mathis Hjort Hjelmsø, Sarah Mollerup, Randi Holm Jensen, Carlotta Pietroni, Oksana Lukjancenko, Anna Charlotte Schultz, Frank M. Aarestrup, Anders Johannes Hansen

**Affiliations:** 1 Research Group for Genomic Epidemiology, Technical University of Denmark, Kongens Lyngby, Denmark; 2 Centre for GeoGenetics, Natural History Museum of Denmark, University of Copenhagen, Copenhagen, Denmark; 3 Division of Microbiology and Production, The National Food Institute, Technical University of Denmark, Kongens Lyngby, Denmark; Oklahoma State University, UNITED STATES

## Abstract

Human viral pathogens are a major public health threat. Reliable information that accurately describes and characterizes the global occurrence and transmission of human viruses is essential to support national and global priority setting, public health actions, and treatment decisions. However, large areas of the globe are currently without surveillance due to limited health care infrastructure and lack of international cooperation. We propose a novel surveillance strategy, using metagenomic analysis of toilet material from international air flights as a method for worldwide viral disease surveillance. The aim of this study was to design, implement, and evaluate a method for viral analysis of airplane toilet waste enabling simultaneous detection and quantification of a wide range of human viral pathogens. Toilet waste from 19 international airplanes was analyzed for viral content, using viral capture probes followed by high-throughput sequencing. Numerous human pathogens were detected including enteric and respiratory viruses. Several geographic trends were observed with samples originating from South Asia having significantly higher viral species richness as well as higher abundances of salivirus A, aichivirus A and enterovirus B, compared to samples originating from North Asia and North America. In addition, certain city specific trends were observed, including high numbers of rotaviruses in airplanes departing from Islamabad. Based on this study we believe that central sampling and analysis at international airports could be a useful supplement for global viral surveillance, valuable for outbreak detection and for guiding public health resources.

## Introduction

Viral infectious diseases are a major burden on human society. Viral pathogens are a very diverse group with recognized species responsible for both gastroenteritis [1], respiratory tract infections [2], hepatitis [3], cancer [4], and numerous other syndromes collectively killing millions each year [5–7]. In addition, an increasing number of re-emerging viruses have been reported, including ebola virus (EboV) [8], SARS [9], MERS [10] and Zika virus [11], causing large and serious epidemics. Effective surveillance systems are critical for outbreak detection and corresponding timely implementation of public health interventions. Additionally, as international trade and travel increases, the need for global surveillance is growing, as viruses do not respect national borders [12,13]. Several transnational pathogen specific viral surveillance networks exist, including NoroNet (http://www.rivm.nl/en/Topics/N/NoroNet) and GISRS [14], covering noroviruses and influenza viruses, respectively. However, the surveillance of the vast majority of viral human pathogens is being organized at a regional or national level, with limited data sharing and cooperation. In addition, many developing countries do not have the infrastructure of doctors, reference laboratories, and health care bodies required for traditional pathogen surveillance, creating large black boxes on the global health map [15].

Several alternative solutions for disease surveillance have been explored, including drug sales [16] and Google search patterns [17], but consistency and sensitivity have been lacking [18,19]. Environmental surveillance has been used as a tool for monitoring the spread of polio for more than 50 years [20], and combined with high throughput metagenomic sequencing could be an attractive and cost effective surveillance strategy for viral pathogens [21]. Metagenomics is an unbiased detection technique allowing for both the detection of known pathogens as well as the discovery of novel viruses [22,23]. However, significant logistic challenges exist for implementing global environmental sampling and metagenomic analysis in a timeframe relevant for producing actionable information.

In 2017, 4 billion passengers travelled by airplane, and this number is expected to rise in the future [24]. This makes airports attractive control points for infectious diseases. In addition, international airports also allow for unique access to human fecal material from all over the world. The feasibility of using toilet waste for global disease surveillance was explored in a previous study, focusing on bacterial pathogens and antimicrobial resistance, with promising results [25]. In addition, this strategy would allow for a high degree of flexibility, with the option of increasing the sampling frequency of incoming airplanes from certain regions, which could be very valuable in outbreak situations.

The aim of this study was to produce and evaluate a method for viral analysis of airplane toilet waste enabling simultaneous detection and quantification of human viral pathogens.

Our protocol, including viral capture probes followed by metagenomic sequencing, was evaluated on airplane sewage from 19 long distance airliners.

## Materials and methods

### Sampling

**Fig 1:**
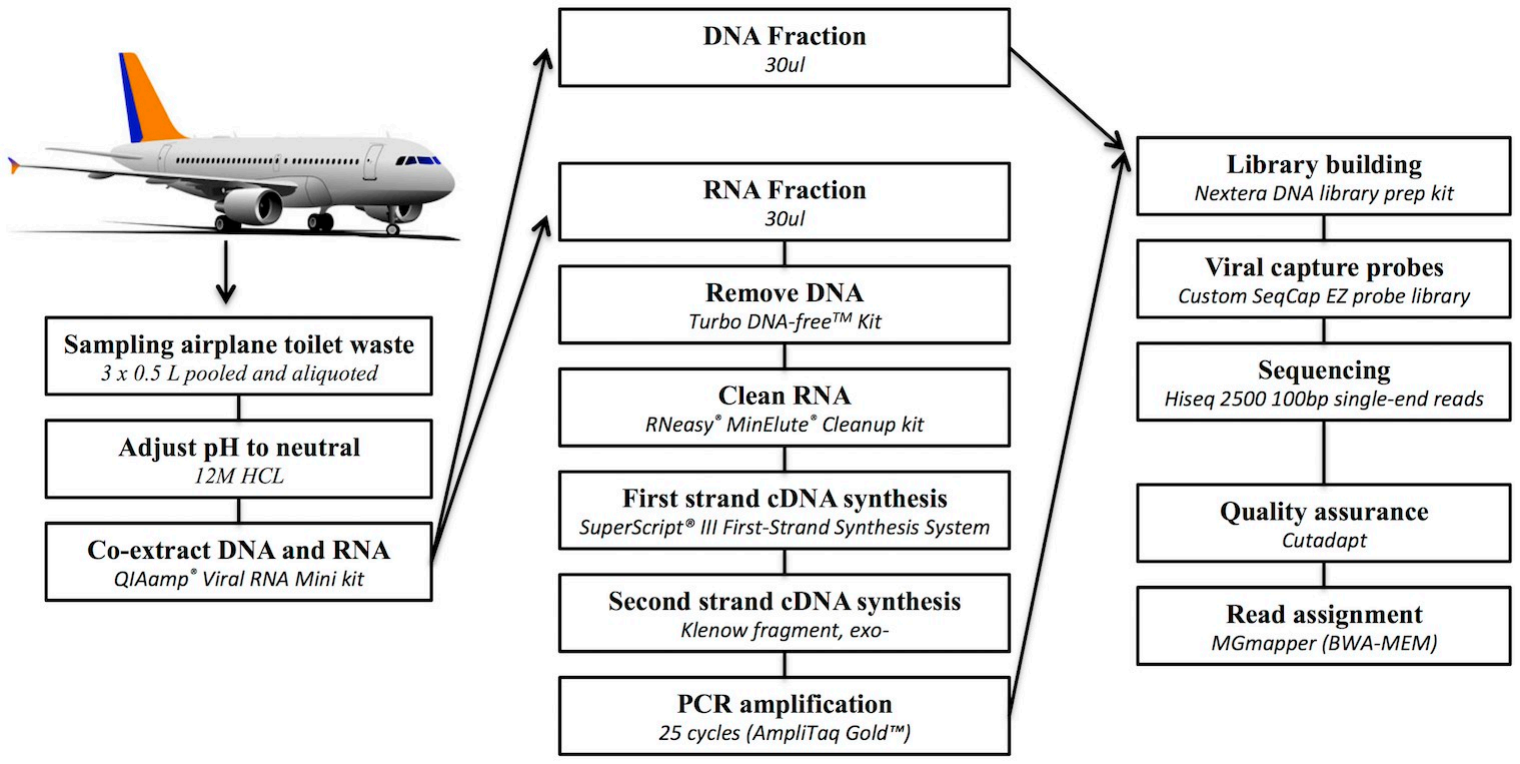
Protocol flowchart. Overview of the different steps in sampling, extraction, cDNA synthesis, library building, sequencing and bioinformatics used in our protocol for the detection and quantification of human viral pathogens from airplane toilet waste.

Sampling was done as previously described [25]. Briefly, airplane toilets were sampled from 19 long distance flights arriving in Copenhagen between June and September 2013, from the nine cities: Bangkok, Beijing, Islamabad, Kangerlussuaq, Newark, Singapore, Tokyo, Toronto, and Washington DC, with permission from the airline (SAS) cleaning service. Three 0.5 L samples of toilet waste were obtained from each airplane, pooled, aliquoted, and stored at -80 °C until nucleotide extraction (Fig 1).

### Nucleic acid extraction

Before extraction the highly basic airplane toilet waste (pH > 11) was pH adjusted to 7 using HCl (12M). 140 μl of airplane toilet waste was used as input for nucleic acid extraction. A positive and negative extraction control was included consisting of a mixture of 8E5 cells carrying one copy of proviral human immunodeficiency virus 1 (HIV-1) genome and human adenovirus (HadV) [26], and sterile molecular grade H_2_O, respectively. DNA and RNA were co-extracted using the QIAamp Viral RNA Mini kit (Qiagen, Valencia CA, USA) according to protocol. The eluate was then split into a RNA and DNA fraction. The RNA fraction was treated with Turbo DNA-free Kit (Invitrogen, USA) to remove DNA, and the remaining RNA purified with the RNeasy MinElute Cleanup kit (Qiagen, Valencia CA, USA). The purified RNA was used as input for cDNA synthesis and subsequent PCR amplification as previously described [27]. Briefly, first strand cDNA synthesis was performed using the SuperScript III First-Strand Synthesis SuperMix (Invitrogen, Carlsbad, California) and 1 μL Primer A (50 μM) (5’-GTTTCCCAGTCACGATCNNNNNNNNN-3’) according to the manufacturer’s instructions. Second strand DNA synthesis was performed using Klenow Fragment exo-polymerase. Double stranded DNA products were PCR amplified using AmpliTaq Gold (Qiagen, Valencia CA, USA) as per manufacturer’s instruction using 0.8 μM Primer B (5′- GTTTCCCAGTCACGATC -3′) and the following conditions, 10 min at 95°C, 25 cycles of amplification (94°C for 30 s, 40°C for 30 s, 50°C for 30 s and 72°C for 1 min), and 1 cycle of elongation (72°C for 10 min). PCR products were purified using the MinElute PCR Purification Kit (Qiagen, Valencia CA, USA).

### Library building, viral enrichment and sequencing

Double indexed sequencing libraries were produced using the Nextera DNA library prep kit (Illumina, CA, USA), with input consisting of a pool of 25 ng of DNA and 25 ng of amplified cDNA for each individual sample. In addition, a negative library control was included using sterile molecular grade H_2_O. The libraries were then enriched for viral sequences using a custom SeqCap EZ probe library designed and synthesized by Roche Nimblegen (CA, USA). The probes were constructed from a reference list consisting of 2,339 viral sequences and genomes from viruses infecting vertebrates downloaded from Genbank in 2014 [28]. The capture reaction was done according to protocol except for the 2 x dilution of Hybridization component A, which allowed for a less stringent hybridization between sample DNA fragments and the viral capture probes, increasing the probe selectivity range [29]. Four to five Nextera libraries were pooled and run together in one capture reaction. After capture, the libraries were sequenced on an Illumina HiSeq 2500 producing 100 bp single-end reads.

### Read processing and alignment

Bioinformatic analysis was done as previously described [27]. Briefly, the quality assurance was done using Cutadapt [30], trimming reads with a Phred score below 20, removing adaptors and subsequent discarding reads shorter than 50 bp. Read assignment was done using the read-alignment software MGmapper (https://cge.cbs.dtu.dk/services/MGmapper/), which is based on the BWA-MEM algorithm run with default settings (ver. 0.7.7-r441) [31,32]. Reads were mapped to a series of freely available reference databases, covering viruses, bacteria and eukaryotes (S1 Table). Reads were mapped in best-mode, meaning that mapping was performed against all databases simultaneously, and later for each read the best hit among all alignments was chosen. A read was considered as a hit only if the alignment score (AS) was above 30 and was higher than the score from the second best alignment (XS). Throughout the mapping approach, only the most reliable hits were accepted i.e. reads were accepted provided that each read maps with an alignment length being at least 70% of the read length. As the single read mapping approach can be unspecific in cases where homologues regions are shared between database entries, virus hits were conservatively reported on the species level, to reduce the chance of misclassification. The raw Illumina sequences are publicly available at the European Nucleotide Archive (ENA) (https://www.ebi.ac.uk/ena/data/view/PRJEB30546).

### Statistics

To test for significant regional differences the 19 airplanes were divided into the three regions; North America (Toronto, Newark, Washington DC, and Kangerlussuaq), South Asia (Singapore, Bangkok, and Islamabad), and North Asia (Tokyo, and Beijing). The viral read counts per million (VRPM) were calculated by normalizing the read count for each specific virus relative to the total viral read count. This was done for each sample as follows: (read count virus A/total viral read count)*10^6^. This normalization accounts for differences in sequencing depth, and removes the influence of variation in bacterial/human reads. As the data were not normally distributed, the non-parametric Kruskal-Wallis test was used to test for significant differences between the three regions. If p < 0.05 additional pairwise Wilcoxon rank sum tests were performed with Bonferroni correction for multiple testing. Heatmaps were done using the R package pheatmap [33]. All statistics were done in R [34].

## Results

**Fig 2:**
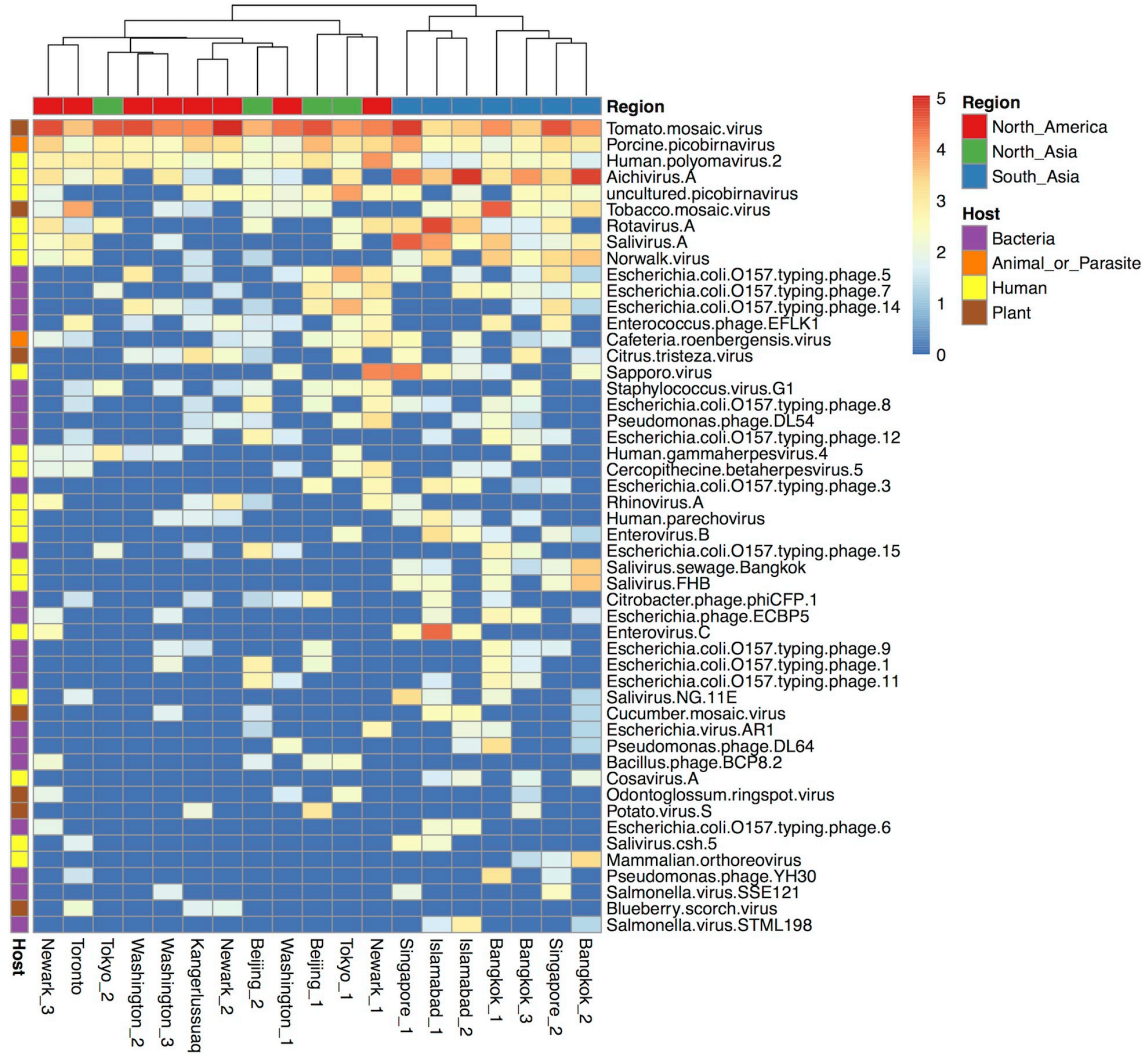
Heatmap of the top 50 most abundant viruses in the airplane toilet waste. Read number values were converted to Viral Reads Per Million (VRPM) and log 10 transformed. Hierarchical clustering was done on the airplane samples and colored by region. The putative viral host is marked in the left column. In some cases the host is debated, and these are discussed more in the Discussion and Strengths and Limitations sections.

The sequencing of the 19 airplane sewage samples produced an average of 19.4 million 100 bp single-end reads per sample. Around 50% of the reads did not map to any of the databases used in this study, which is in line with previous studies [27,35,36], whilst the majority of assigned reads mapped to bacterial databases (S1 Fig). Only an average of 0.24% of the reads mapped to viruses, and 0.01% to human viruses despite the use of the viral capture probes. The viral reads mapped to 287 different viral strains from the viral databases, but due to the high chance of misclassification using read aligners we conservatively agglomerated all reads on the species level. This resulted in a total of 104 viral species, from 31 different viral families (Fig 2). Of these species 37 had a confirmed or suspected human host, 6 were parasite or animal viruses, 12 were plant viruses and 49 were bacteriophages.

### Sequencing quality control

Sequencing reads mapping to HAdV, EboV and HIV were detected in the negative control and these species were conservatively removed from the dataset prior to further analysis [37]. The presence of a large number of reads mapping to EboV in the positive control were investigated by coverage analysis, elucidating that all reads mapped to the same short region of the EboV reference genome (S2 Fig). When blasting that region against the NCBI nr database, the top hits included both EboV and HIV-1, suggesting that the presence of EboV reads were a product of misclassification during read assignment due to a homologous region shared by the two viruses.

In addition, the negative controls also had large numbers of bacterial *Thermus sp*. reads, most likely being a DNA contaminant of the used *Taq* polymerases as this protein was originally isolated form *Thermus aquaticus YT-1* [38], stressing the importance of the inclusion and analysis of negative controls to reduce false positives [39].

### Evaluation of protocol for viral detection and quantification

Several viral families with human pathogens were detected in the airplane toilet waste including *Picornaviridae*, *Caliciviridae*, *Polyomaviridae*, *Reoviridae*, and *Picobirnaviridae*, with a total of 37 confirmed or putative human viral pathogens. However, more than 90% of the viral reads mapped to the viral families *Myoviridiae* and *Virgaviridae* (S3 Fig), consisting of bacteriophages and plant pathogens, respectively.

**Fig 3:**
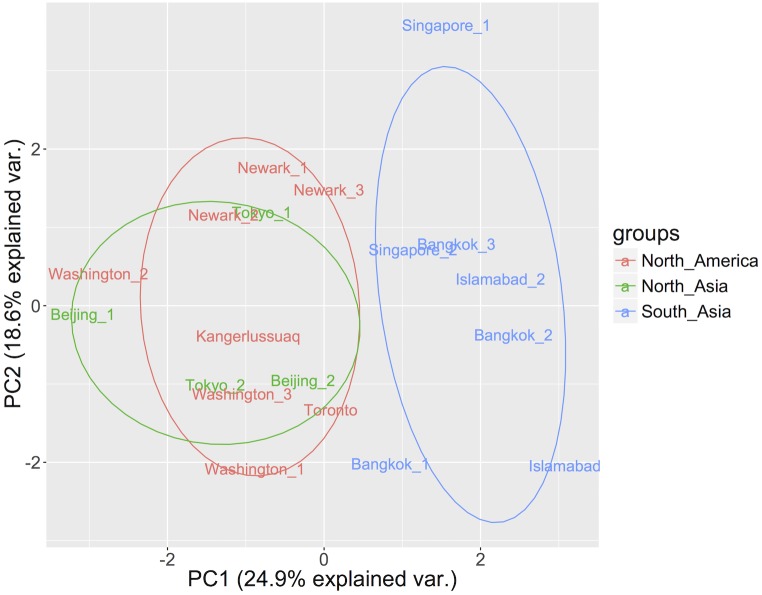
Principal component analysis (PCA) of the airplane sewage viral community composition. Log10 transformed viral reads per million (VRPM) values for the 12 most abundant virus families were used as input. The individual samples are colored according to their assigned region.

When studying patterns in the viral community composition, the sewage samples arriving from South Asia generally had higher abundances of caliciviruses, reoviruses and picornaviruses, resulting in the separate clustering of these samples in a PCA plot (Fig 3).

**Fig 4:**
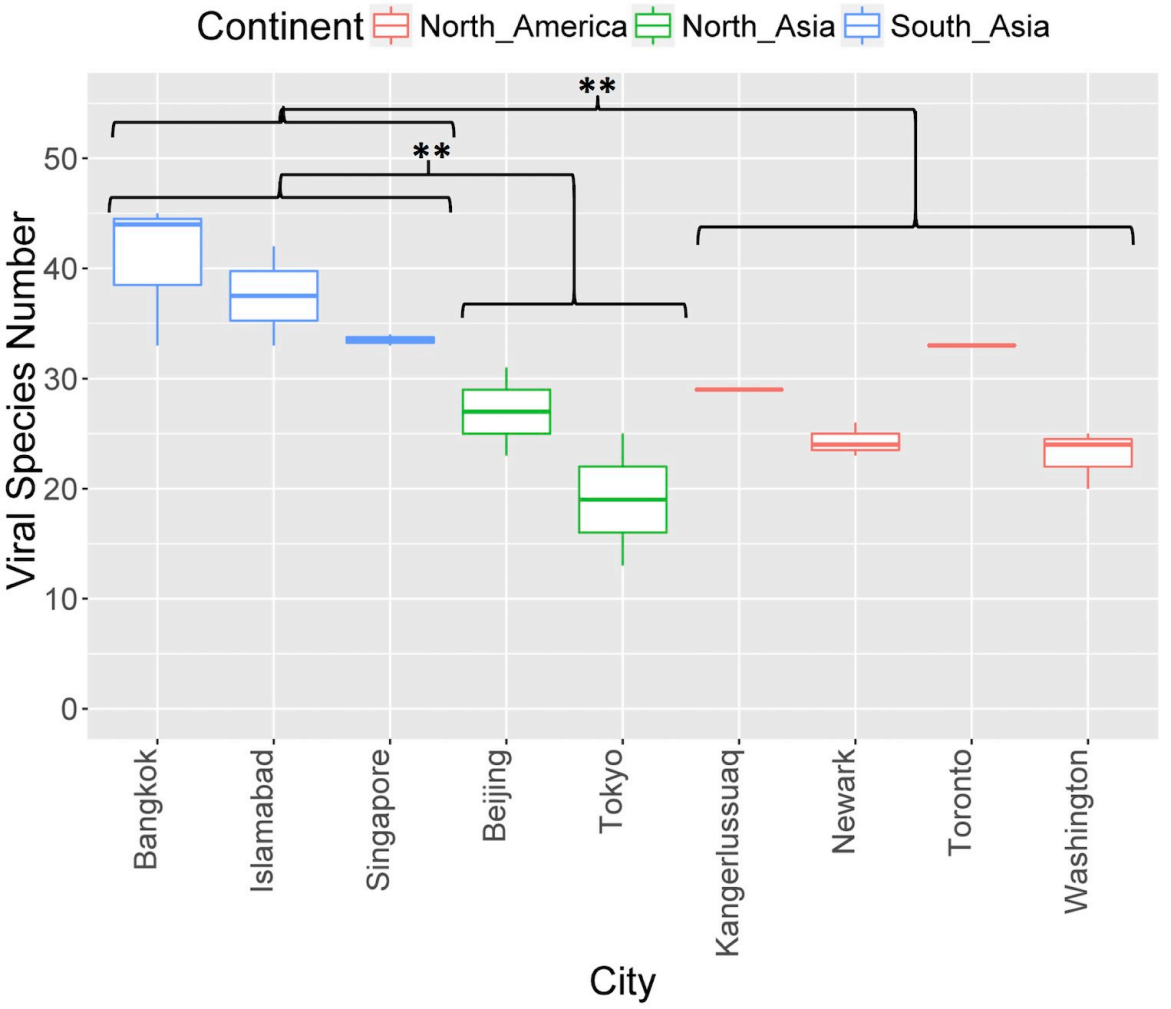
Viral species richness of the samples grouped according to their city of departure. Statistical analysis was done on region level, using Anova to test for significance (p<0.05) followed by pairwise t-tests with Bonferroni correction for multiple testing. ** = p<0.01.

In addition, samples from South Asia also had significantly higher species richness than both North America and North Asia (t-test, p<0.01) (Fig 4).

**Fig 5:**
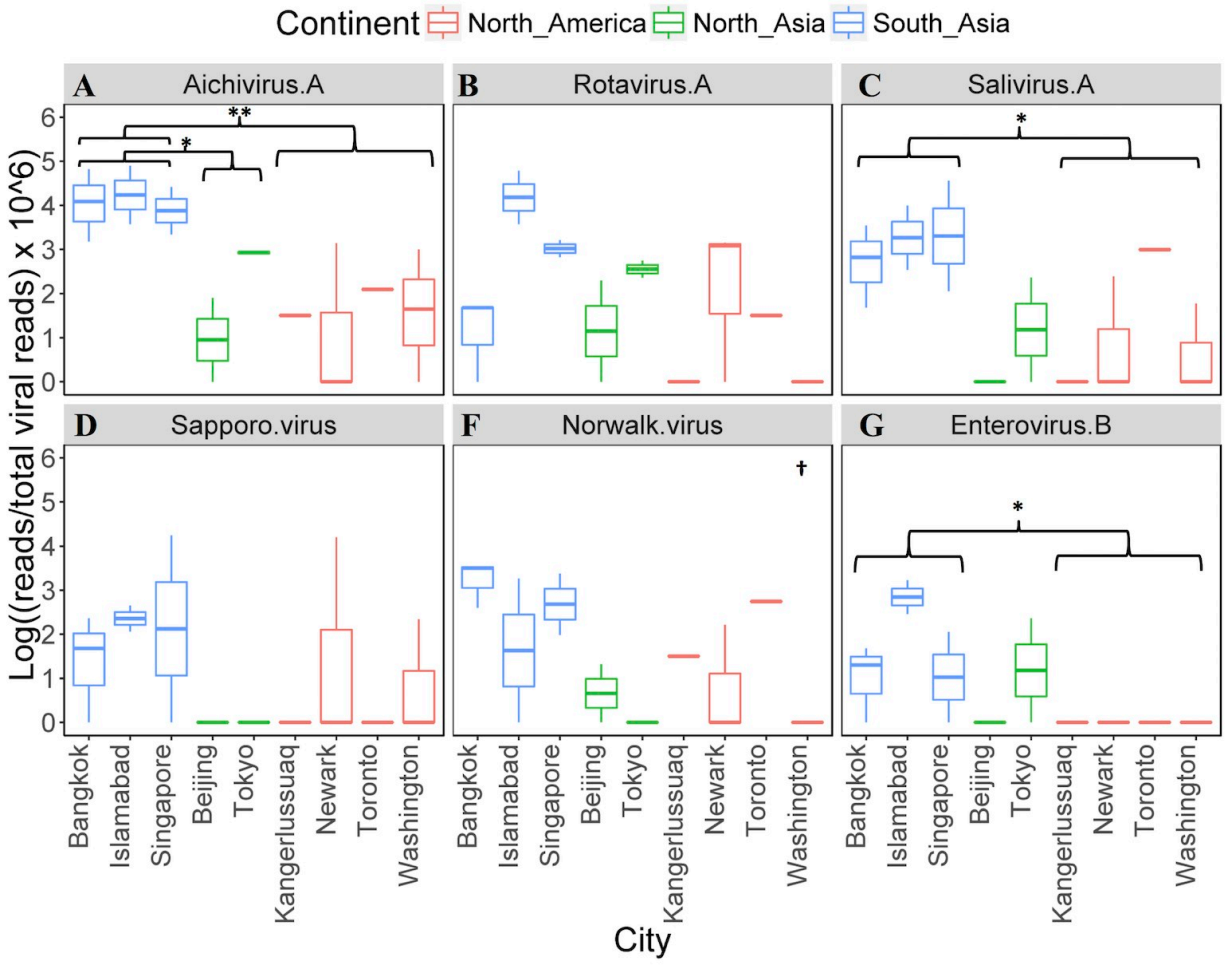
Enteric viruses detected in the sewage from the 19 airplanes. The airplane samples are grouped according to their departing city (n = 1–3). The y-axis was log-transformed viral reads per million (VRPM). For the statistical analysis, the cities were grouped according to their assigned region, and tested for significance using the Kruskal-Wallis rank sum test. If significant, the pairwise significance was determined using the Wilcoxon test with Bonferroni correction for multiple testing. * = p-Wilcoxon < 0.05, ** = p-Wilcoxon < 0.01, † = p-Kruskal-Wallis < 0.05.

Several enteric viruses were detected in the sewage material, and they were generally more abundant in the samples from South Asia (Fig 5). This difference was statistically significant when compared with samples originating from North America for aichivirus A, salivirus A and enterovirus B (Fig 5A, 5C and 5G).

In this pilot study, we observed that some viruses were found consistently in samples from specific cities, for example rotavirus A in airplanes departing from Islamabad, which could suggest a high endemic presence. However, our findings are sensitive to stochastic events and transit passengers due to the low number of samples in this study, and replication is needed to confirm our observations.

Not only enteric viruses were detected in the sewage but also the respiratory rhinovirus A, and the latent skin infection gammaherpesvirus 4, better known as Epstein-Barr virus (Fig 2). Some viruses were detected in all samples including human polyomavirus 2, better known as JC polyomavirus (JCV), and the veterinary pathogen porcine picobirnavirus (PBV) (Fig 2).

To study the sensitivity of the metagenomic approach, linear regression analysis was done on the reads mapping to the norovirus (NoV) GII genome together with qPCR generated genome copy numbers procured from a previous study using the same samples [25]. A strong association between the two factors was observed (R^2^ = 0.58), indicating that our metagenomic analysis could be used as a semi-quantitative measure of viruses in the sample, although the qPCR method seemed to be more sensitive (S4 Fig).

## Discussion

In this study, toilet waste from 19 airplanes was subjected to metagenomic virome analysis. Contamination of some of the samples, including the negative controls, with HAdV and HIV from the positive control were observed. This contamination could have happened in the laboratory, despite working in a dedicated viral clean lab [40], but is more likely a sequencing artifact [26,41]. Because of the large number of PCR cycles for cDNA amplification, library building, and capture, this type of analysis is very vulnerable to cross contamination, and the inclusion of negative controls should be considered obligatory [37]. False positives, as were identified in this study, are a big challenge for diagnostics and metagenomic surveillance surveys and have been reported previously in studies of both bacteria and viruses [42,43].

NoV GII quantification, using our metagenomics approach, showed good correlation with previous qPCR analysis (S4 Fig), suggesting that our protocol can be used for quantifying the viral burden in the sewage samples. A similar relationship was found in a previous study testing eight different viruses, further supporting the quantitative capabilities of viral metagenomics [22].

Despite the low number of samples in this study we observed regional differences in the viral community composition, with the samples originating from South Asia clustering separately from the ones from North America and North Asia (Fig 3). This finding is supported by a recent study detecting national differences in viral exposure [44]. The reason for this geographical difference is unknown to the authors, but could include factors such as diet, socioeconomic status, host factors, genetics, climate, drinking water quality, sanitary conditions and hygiene standards [45]. However, airplane passengers might not be accurate representatives of the residents in the city of origin, which should be considered in the interpretation. Most of the detected viral human pathogens were enteric and generally were found in highest abundance in the airplanes coming from South Asia. Aichivirus A was one of these, and has been associated with a wide range of clinical illnesses including diarrhea, vomiting, fever, purulent conjunctivitis, and respiratory symptoms [46]. Furthermore, it has previously been detected in cases of gastroenteritis in Pakistani children and in Japanese airplane passengers returning home from South East Asia [47], supporting the notion of a high prevalence of aichivirus in this region. Salivirus A, another member of the family *Picornaviridae*, was also prevalent in samples from South Asia. This virus has been associated with gastroenteritis [48] but its pathogenicity has not been fully resolved as other studies have found it in equal numbers in both healthy and sick children [49]. However, widespread occurrence of salivirus in humans has been confirmed [50,51] as well as previous detection in sewage [52].

Islamabad, the city of departure with highest rotavirus abundances, and the rest of Pakistan, have huge problems with rotavirus infections with millions of cases and an estimated 14,700 deaths of children below 5 years of age every year [53]. Intervention strategies include vaccination and in January 2017 (after the samples were collected) rotavirus vaccination became a part of the immunization program in Pakistan. We argue that airplane surveillance could be an excellent tool for producing directly comparable surveillance data across national borders, to identify areas with high occurrence of viral infection and disease, and guide public health intervention strategies. Enteric viruses cause high numbers of gastroenteritis globally and are responsible for hundreds of thousands of deaths each year in the developing world [53,54]. We believe that national comparisons, such as this pilot study, could help increase awareness in both local and international public health departments and lead to increased disease intervention efforts.

Human JCV was found in all airplane samples. JCV has been reported in all parts of the world with seroprevalence rates of 65–90% as well as a high rate of viral excreters through urine [55]. In addition, JCV has been proposed as a bioindicator for human fecal contamination [56], and this study confirms global high levels of JCV in human waste.

In this study, results from the viral analysis of airplane toilet material was finished several days after landing, too late to isolate infected passengers. However, future development in sequencing techniques might allow for analysis of air or toilet material during flight [57], allowing for screening and quarantine of passengers infected with high-risk viruses at the border [58].

### Strengths and limitations

Limitations of this study include the low number of samples representing both individual cities and larger regions, making baseline values and trends sensitive to stochastic events. However, the intercontinental airplanes used in this study have room for 245–262 passengers, which in other contexts would be a quite respectable sample size, especially if sampling was done more frequently than in this study. Another limitation is the possibility of transit passengers, which could interfere with the signal from individual cities, especially in small sample studies such as this one. Airplane passengers might also not be representative of the average citizen in all countries, with could serve as a bias. It is also a limitation that we do not know the clinical history of the airplane passengers at the time of flight, which would have been very useful to validate our viral findings.

To enrich for pathogenic viruses, we used a custom library of viral capture probes targeting vertebrate pathogens. Viral capture probes have previously been showed to increase the number of viral reads of up to 3 orders of magnitude [59], and by lowering the stringency of the probe hybridization, as was done in this study, viral sequences not included in the probe design can also be detected [29]. However, most viruses are still undiscovered and not present in current databases [60], and thus not included in the SeqCap probe design, which limits our analysis to only known and already sequenced viruses. Furthermore, the use of capture probes also increases the length of the sample processing with several days, and physical viral enrichment using filters and nucleases might be a faster and more appropriate alternative for real time surveillance [27]. As we use toilet waste in this protocol, we are limited to detecting viruses excreted in the feces or urine, missing important respiratory viruses such as influenza viruses. However, viral shedding in feces has been detected in emerging viral pathogens such as severe acute respiratory coronavirus (SARS-Cov) and ebola virus [61,62], suggesting that they may be possible targets of our protocol. Finally, environmental metagenomic studies, such as this one, with low abundances of target organisms and corresponding low read counts, are very vulnerable to read misclassifications [42]. This was also identified in this study, due to the inclusion of both positive and negative extraction controls. However, our method does not guarantee that no other cases of misclassification exist in our dataset and interpretations should therefore take this into account. One possible example is the presence of the veterinary porcine picobirnavirus in all of our airplane sewage samples, which based on the nature of our samples, is possibly a human strain with no representative in the viral databases. Porcine and human picobirnaviruses can be almost identical and require targeted approaches to discriminate [63]. Alternatively, the detected picobirnavirus could actually be a phage, as has been suggested in two recent publications based on the enrichment of functional prokaryotic ribosome binding sites in picobirnavirus genomes [64,65]. Assembly based bioinformatic methods have the potential to reduce the misclassification problem, increasing the specificity of viral assignment, but the low number of viral reads in our samples in combination with a focus on speed made this option unfeasible. However, assembly based assignment should be pursued in further studies of viral surveillance in environmental samples. As we used a reference based approach our results are also limited by the current state of viral sequence databases, including only a small part of the viral diversity.

## Conclusion

In conclusion, our protocol was able to detect and quantify enteric, respiratory, and latent viruses in toilet waste from 19 international flights arriving in Copenhagen Airport using metagenomics. Several viruses were found in significantly higher quantities in samples arriving from South Asia, including salivirus A, aichivirus A and enterovirus B, and the samples from this region had a higher viral species richness. In addition, planes from certain cities were identified as having high amounts of rotaviruses and NoVs in samples taken months apart. However, an increase in sensitivity and specificity is needed before our approach can be implemented by public health professionals, which could be overcome by further development in nucleotide extraction and bioinformatics analysis. With improvements, surveillance of viral particles in airplane toilet waste using metagenomics could be a valuable addition to current surveillance efforts, producing global comparable surveillance data relevant for outbreak detection and implementation of public health interventions.

## Supporting information

S1 Fig Sequence read origin on the kingdom level from the individual airplane samples and experimental controls. Individual reads were mapped against the databases shown in S1 Table, and results from the multiple bacterial and viral databases added together before plotting. NC = negative extraction control (H2O), PC = Positive control (HAdV and HIV), LibraryNC = library negative control (H2O).
(PDF)

S2 Fig Coverage plot of the three viruses found in the positive control. Reads were mapped to the reference genomes (A) >NC_001405.1 Human adenovirus C, (B) >NC_001802.1 Human immunodeficiency virus 1, and (C) KC242800 gb Zaire ebolavirus isolate EBOV.
(TIF)

S3 Fig Viral family abundances of the individual airplane samples and experimental controls. Only the 10 most abundant families are shown. NC = negative extraction control (H2O), PC = Positive control (HAdV and HIV), LibraryNC = library negative control (H2O).
(PDF)

S4 Fig NGS and qPCR correlation. Correlation between Norovirus GII qPCR genome copies and NGS reads (Viral Reads Per Million). Both the qPCR and NGS data were log transformed prior to the plotting and linear regression analysis.
(PDF)

S1 Table Overview of reference sequence databases and associated download information. (PDF)
