# Supplementary figures and images for: Metagenomic analysis of viruses in toilet waste from long distance flights—A new procedure for global infectious disease surveillance
Source: PLoS One. 2019 Jan 14;14(1):e0210368. doi: 10.1371/journal.pone.0210368 (PMC6331095; doi:10.1371/journal.pone.0210368)

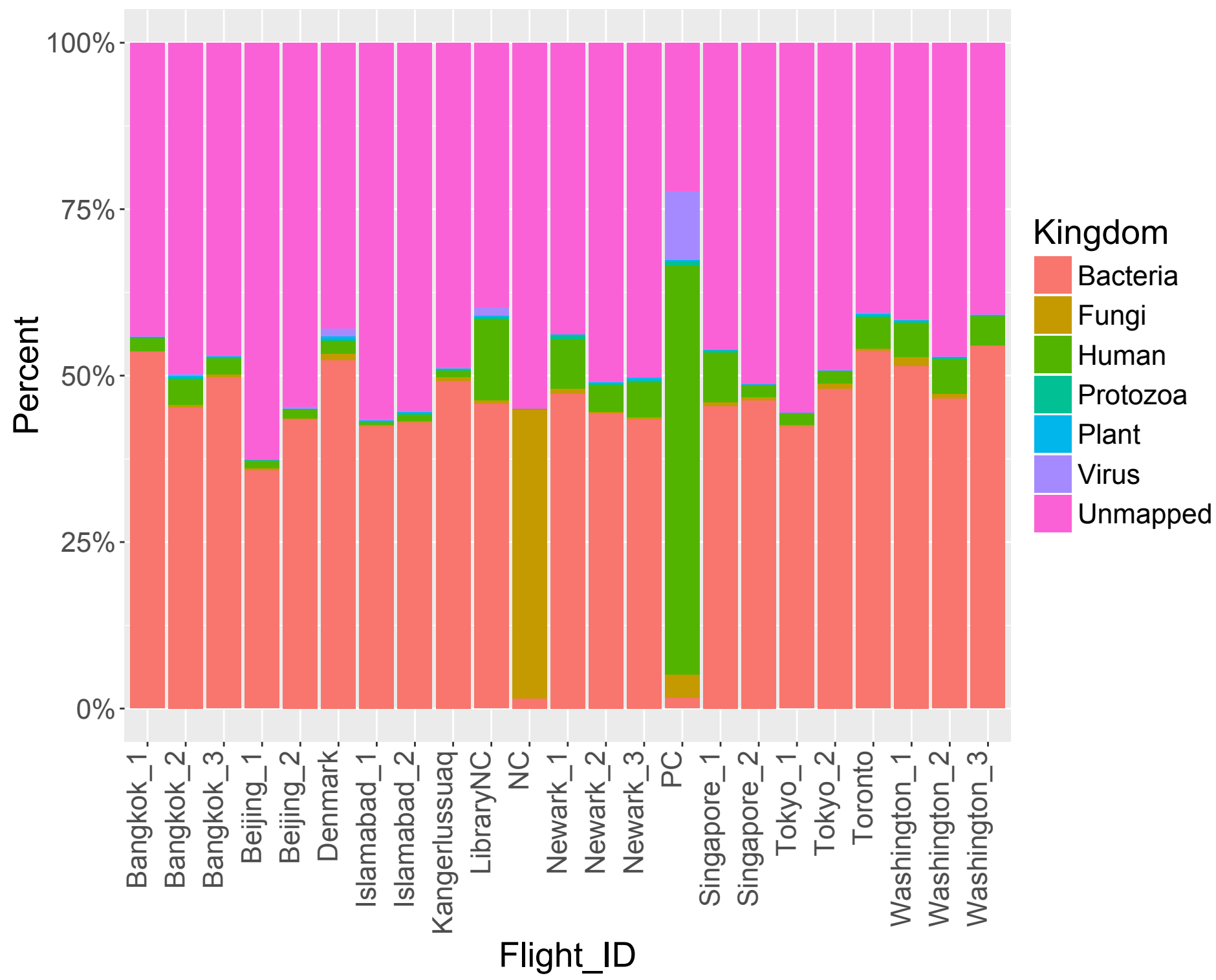

Supplement: S1 Fig — Individual reads were mapped against the databases shown in S1 Table, and results from the multiple bacterial and viral databases added together before plotting. NC = negative extraction control (H2O), PC = Positive control (HAdV and HIV), LibraryNC = library negative control (H2O). (PDF) [file pone.0210368.s001.pdf]

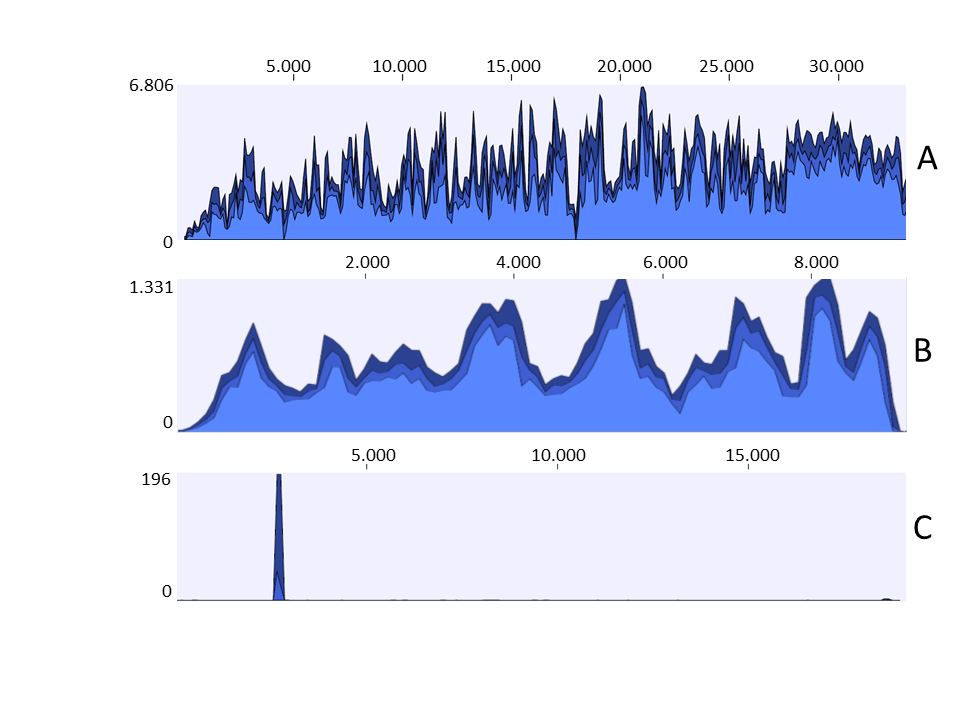

Supplement: S2 Fig — Reads were mapped to the reference genomes (A) >NC_001405.1 Human adenovirus C, (B) >NC_001802.1 Human immunodeficiency virus 1, and (C) KC242800 gb Zaire ebolavirus isolate EBOV. (TIF) [file pone.0210368.s002.tif]

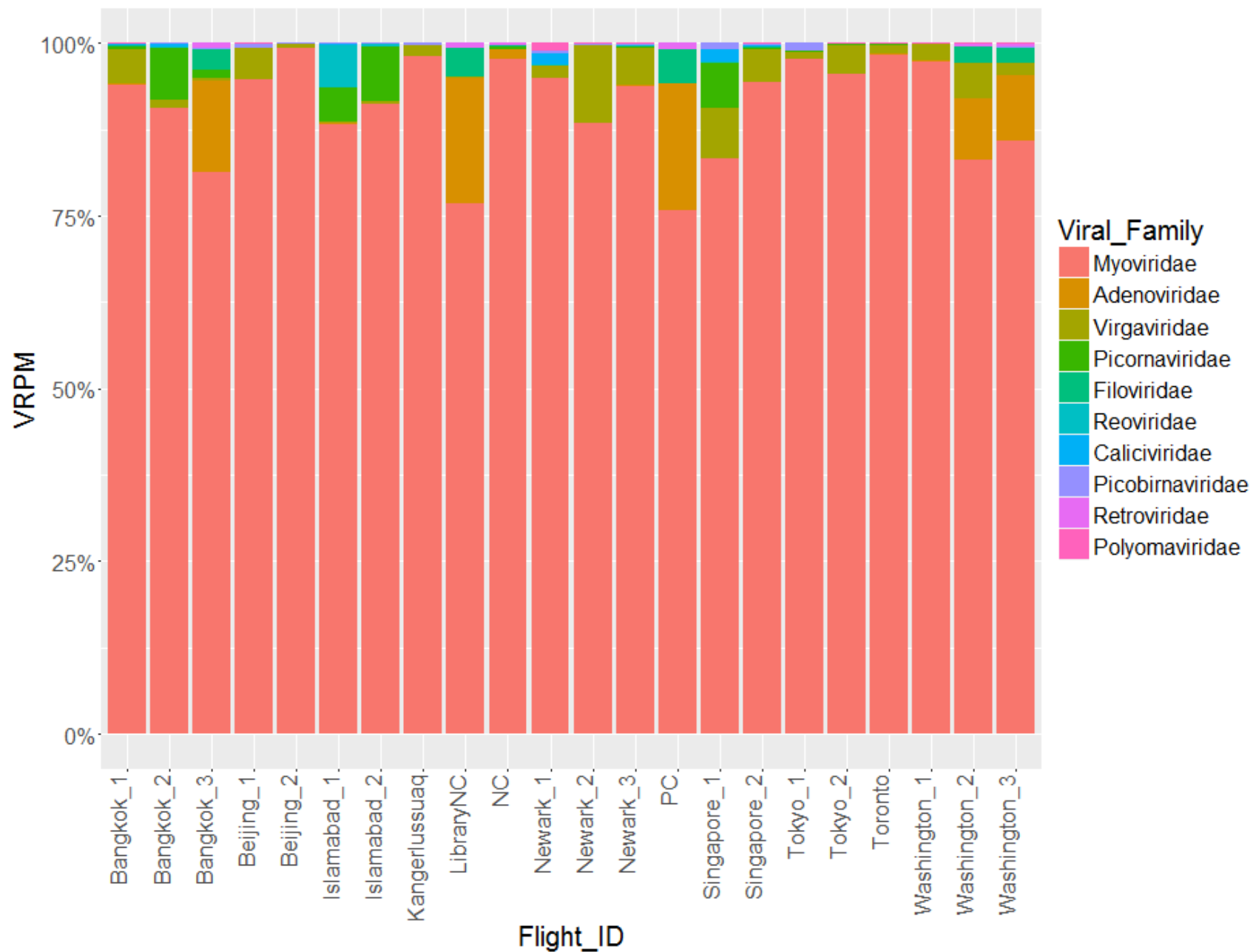

Supplement: S3 Fig — Only the 10 most abundant families are shown. NC = negative extraction control (H2O), PC = Positive control (HAdV and HIV), LibraryNC = library negative control (H2O). (PDF) [file pone.0210368.s003.pdf]

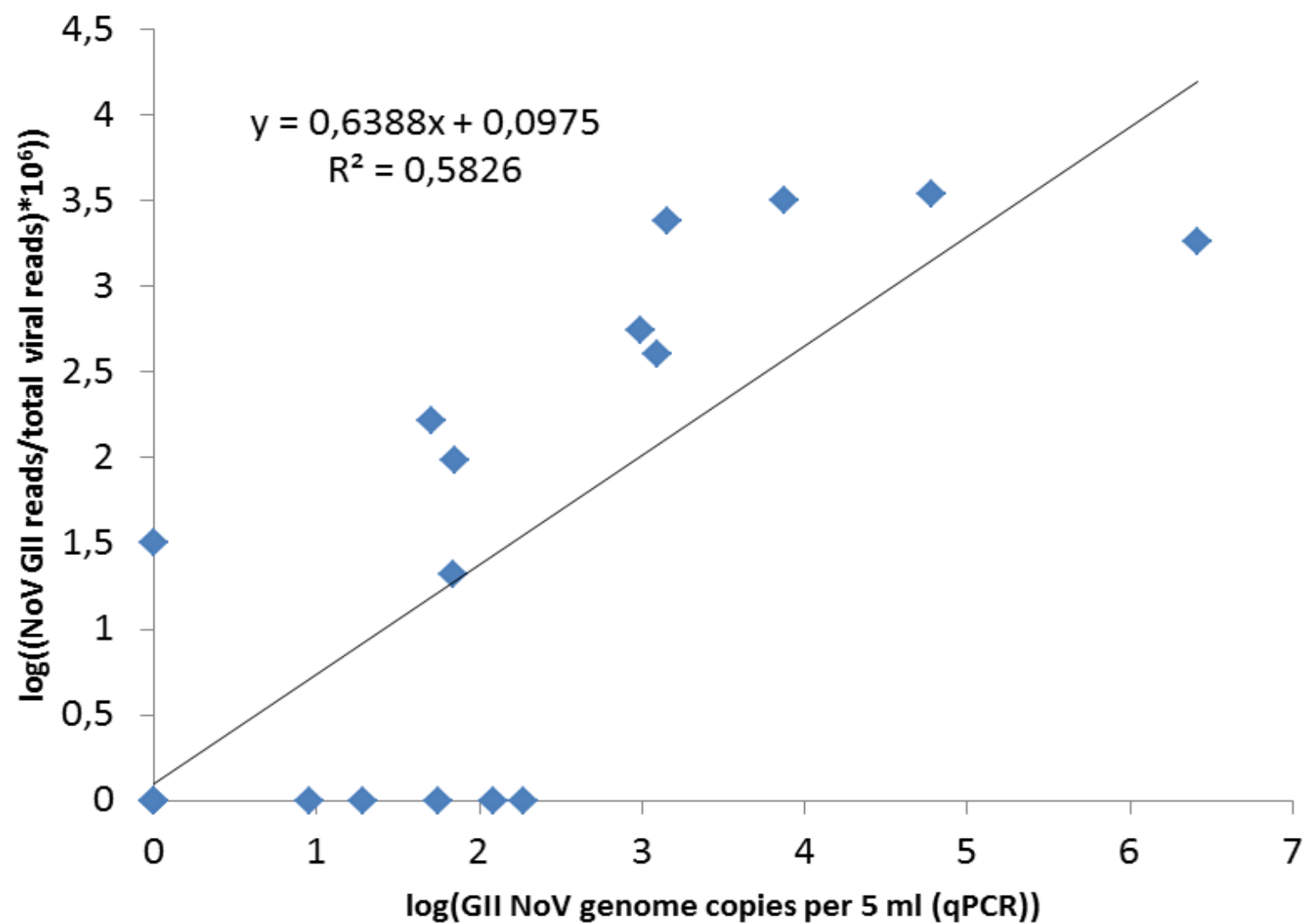

Supplement: S4 Fig — Correlation between Norovirus GII qPCR genome copies and NGS reads (Viral Reads Per Million). Both the qPCR and NGS data were log transformed prior to the plotting and linear regression analysis. (PDF) [file pone.0210368.s004.pdf]
