# Supplementary material for: Metagenomic analysis of viruses in toilet waste from long distance flights—A new procedure for global infectious disease surveillance
Source: PLoS One. 2019 Jan 14;14(1):e0210368. doi: 10.1371/journal.pone.0210368 (PMC6331095; doi:10.1371/journal.pone.0210368)
Supplement: S1 Table — (PDF) [file pone.0210368.s005.pdf]

Database name	Download information
**HumanMicrobiome**[2]	https://www.ncbi.nlm.nih.gov/bioproject/28331
**MetaHitAssembly**[2]	http://www.ncbi.nlm.nih.gov/nuccore/?term=PRJEB674 (PRJEB674 - PRJEB1046)
**Bacteria**[2]	ftp://ftp.ncbi.nih.gov/genomes/genbank/bacteria
**Human**	http://www.ncbi.nlm.nih.gov/assembly/GCF_000001405.25/#/def_asm_Primary_Assembly<br>Human genome ver. GRCh37.p13
**Protozoa**	ftp://ftp.ncbi.nlm.nih.gov/ genomes/genbank/protozoa
**Virus**[1]	ftp://ftp.ncbi.nih.gov/genomes/virus
**Virus_NCBI**[1]	http://www.ncbi.nlm.nih.gov/genbank/
**Virus_Vipr**[1]	http://www.ncbi.nlm.nih.gov/pmc/articles/PMC3245011
**Plants**	ftp://ftp.ncbi.nlm.nih.gov/ genomes/genbank/plant
**Fungi**	ftp://ftp.ncbi.nlm.nih.gov/ genomes/genbank/fungi

[1] All viral databases were combined into one. [2] All bacterial databases were combined into one. Reference sequence information can be obtained from the URL's shown in 'Download information'.
